# A novel proline-rich M-superfamily conotoxin that can simultaneously affect sodium, potassium and calcium currents

**DOI:** 10.1590/1678-9199-JVATITD-2020-0164

**Published:** 2021-06-11

**Authors:** Manyi Yang, Yubin Li, Longfei Liu, Maojun Zhou

**Affiliations:** 1 Department of Hepatobiliary and Pancreatic Surgery, NHC Key Laboratory of Nanobiological Technology, Xiangya Hospital, Central South University, Changsha, Hunan, China.; 2 Department of Oncology, State Local Joint Engineering Laboratory for Anticancer Drugs, NHC Key Laboratory of Cancer Proteomics, Xiangya Hospital, Central South University, Changsha, Hunan, China.; 3 Department of Urology, National Clinical Research Center for Geriatric Disorder, Xiangya Hospital, Central South University, Changsha, Hunan, China.

**Keywords:** Conotoxin, Conopeptide, Sodium currents, Potassium currents, Calcium currents

## Abstract

**Background:**

Conotoxins have become a research hotspot in the neuropharmacology field for their high activity and specificity in targeting ion channels and neurotransmitter receptors. There have been reports of a conotoxin acting on two ion channels, but rare reports of a conotoxin acting on three ion channels.

**Methods:**

Vr3a, a proline-rich M-superfamily conotoxin from a worm-hunting *Conus varius*, was obtained by solid-phase synthesis and identified by mass spectrometry. The effects of synthesized Vr3a on sodium, potassium and calcium currents were tested on rat DRG cells by patch clamp experiments. The further effects of Vr3a on human Ca_v_1.2 and Ca_v_2.2 currents were tested on HEK293 cells.

**Results:**

About 10 μM Vr3a has no effects on the peak sodium currents, but can induce a ~10 mV shift in a polarizing direction in the current-voltage relationship. In addition, 10 μM Vr3a can increase 19.61 ± 5.12% of the peak potassium currents and do not induce a shift in the current-voltage relationship. An amount of 10 μM Vr3a can inhibit 31.26% ± 4.53% of the peak calcium currents and do not induce a shift in the current-voltage relationship. The IC_50_ value of Vr3a on calcium channel currents in rat DRG neurons is 19.28 ± 4.32 μM. Moreover, 10 μM Vr3a can inhibit 15.32% ± 5.41% of the human Ca_v_1.2 currents and 12.86% ± 4.93% of the human Ca_v_2.2 currents.

**Conclusions:**

Vr3a can simultaneously affect sodium, potassium and calcium currents. This novel triple-target conotoxin Vr3a expands understanding of conotoxin functions.

## Background

Conotoxins or conopeptides are marine bioactive peptides derived from more than 700 Cone snails [1,2]. Typical conotoxins contain 10-30 amino acids with multiple disulfide bonds and post-translational modifications [3,4]. Due to the high specificity and selectivity of conotoxins in targeting ion channels and neurotransmitter receptors, conotoxins have become a research hotspot in the neuropharmacology field [5]. One of the calcium channel inhibitors, ω-conotoxin MVIIA (ziconotide), was proved by the United States Food and Drug Administration (FDA) in 2004 for treating intractable pain [6]. Moreover, conotoxins can also be used as molecular probes for ion channel research and drug leads for the therapy of pain, addiction, cardiovascular, epilepsy, cancer and so on [7-9].

M-superfamily is one of the biggest superfamilies and has been found in all the *Conus* species tested so far [10]. M-superfamily conotoxins mainly contain a Framework III cysteine pattern (CC-C-C-CC) and a highly homologous signal region [11]. Most of the known active M-superfamily conotoxins are μ-conotoxins that block sodium channels with high potency and subtype selectivity [12]. Due to the high inhibitory activity of rat tetrodotoxin-resistant sodium currents [13], μ-conotoxin SIIIA was used as a preclinical lead for the treatment of pain [14]. μ-conotoxins can also be used as sodium channel research probes for their high subtype selectivity, and may be used as neuroprotective agents against hypoxia or oxidative stress [15]. In addition to these μ-conotoxins, several other M-superfamily conotoxins were found to inhibit nicotinic acetylcholine receptors or block voltage-gated potassium channels [10,16]. M-superfamily conotoxins are further divided into the M-1, M-2, M-3 M-4 and M-5 branch conotoxins, based on the number of amino acids that exist between the fourth and the fifth cysteines [10]. M-1 branch conotoxins have the disulfide connectivity of 1-5, 2-4, 3-6; M-2 branch conotoxins have the disulfide connectivity of 1-6, 2-4, 3-5; while M-4 and M-5 branch conotoxins have the disulfide connectivity of 1-4, 2-5, 3-6 [10].

In our previous study, a specific group of M-superfamily conotoxins was found from a worm-hunting *Conus varius* [17]*.* One of these M-superfamily conotoxins, Vr3a, has no sequence homology with other conotoxins, indicating that it may has a specific physiological function. In this study, we tried to figure out the physiological target of Vr3a. The disulfide connectivity of Vr3a was set as 1-4, 2-5, 3-6 according to the disulfide connectivity of other M-4 conotoxins. The Vr3a peptide was obtained by solid-phase synthesis and identified by mass spectrometry. The sodium, potassium and calcium channel physiological activities of Vr3a were tested on rat DRG cells by patch clamp experiments. The further human Ca_v_1.2 and Ca_v_2.2 physiological activities of Vr3a were tested on HEK293 cells. 

## Methods

### Synthesis of conotoxin Vr3a

Conotoxin Vr3a, whose amino acid sequence is QGCCPPGVCQMAACNPPPCCP, was synthesized by solid-phase polypeptide synthesis. Briefly, conotoxin peptide was assembled on Rink-resin using Fmoc-strategy according to its amino acid sequence. The amino acid residues were coupled using HOBt/HBTU/DIPEA (1:1:0.9) for 2 h and cleavaged from the resin with regent R (90% TFA: 5% thioanisole: 3% 1, 2-ethanedithiol: 2% anisole). Six cysteines were protected by acetamidomethyl (Acm) (Cys3 and 14), methoxytriphenyl (Mmt) (Cys4 and 19) or Triphenylmethyl (Trt) (Cys9 and 20) separately and the three disulfide bonds (3-14, 4-19, 9-20) were successively formed by oxidation. The final oxidized peptide was purified through a C18 reverse phase column (4.6mm×250mm, 5_m particle diameter, 300 Å) on 600E HPLC system (Waters, America). The mobile phase used a gradient of 5-50% solvent B in 30min, where solvent A was H2O/0.1% TFA and solvent B was 100% CH3CN/0.1% TFA. The flow rate was 1 ml/min and the absorbance was monitored at 215 nm. The peptide (in 30% acetonitrile/0.1% TFA) was treated with α-cyano-4-hydroxycinnamic acid (CHCA) and the molecular weight was determined by matrix assisted laser desorption ionization mass spectrum (MALDI-MS) using a REFLEX III time-of-flight mass spectrometer (Bruker Daltonics, America). 

### Acute separation of DRG cells

The animal protocols used in this study were evaluated and approved by the Xiangya Hospital Medical ethics committee of Central South University. Sprague-Dawleys (SD) rats were purchased from Hunan SJA Laboratory Animal Co. Ltd. with a quality license. Acutely separated dorsal root ganglion (DRG) cells were prepared from 30-day-old SD rats of either sex [18]. The rats were euthanized and the dorsal root ganglia tissue was removed quickly and cut into small pieces. The ganglia were treated with collagenase (Sigma, USA) followed by trypsin (Sigma, USA). Trypsin inhibitor (Sigma, USA) was added to a final concentration of 1.5 mg/mL to terminate the enzymatic treatment. After centrifugation, the DRG cells were suspended in essential Dulbecco's modified Eagle's medium (DMEM) with 10% fetal bovine serum and incubated at 37 °C in a CO_2_ incubator for 3 h. Medium DRG cells (diameters 20~30 μm) were used to record TTX-sensitive and TTX-resistant mixed currents.

### Transfection of Ca_v_1.2 and Ca_v_2.2 into HEK293 cells

HEK293 cells, which were obtained from the Cell Bank of the Chinese Academy of Sciences (Shanghai, China) with STR Authentication, were cultured in DMEM with 10% fetal bovine serum. Plasmids of human Ca_v_1.2 and Ca_v_2.2 were cloned by our lab and were separately transfected into HEK293 cells using Lipofectamine 3000 (Invitrogen).

### Intracellular and extracellular solutions for patch clamp

For recording sodium currents, the intracellular solution contained the following composition: 10 mM CsCl, 5 mM NaCl_2_, 10 mM HEPES, 2 mM Mg-ATP, 135 mM CsF, 5 mM EGTA, pH = 7.2 (CsOH), and the extracellular solution contained the following composition: 22 mM NaCl, 110 mM Choline-Cl, 5 mM D-glucose, 10 mM HEPES, 0.8 mM MgCl_2_, 1.8 mM CaCl_2_, pH = 7.4 (NaOH).

For recording potassium currents, the intracellular solution contained the following composition: 120 mM KCl, 1 mM MgCl_2_, 5 mM EGTA, 14 mM Phoshocreatine disodium salt, 5 mM Na_2_-GTP, pH = 7.2 (KOH), and the extracellular solution contained the following composition: 1.8 mM CaCl_2_, 135 mM Choline-Cl, 10 mM D-glucose, 10 mM HEPES, 1 mM MgCl_2_, 4.5 mM KCl, pH = 7.4 (KOH).

For recording calcium currents, the intracellular solution contained the following composition: 120 mM CsCl, 1 mM MgCl_2_, 10 mM HEPES, 4 mM Mg-ATP, 0.3 mM Na_2_-GTP, 10 mM EGTA, pH = 7.2 (CsOH), and the extracellular solution contained the following composition: 140 mM TEA-Cl, 2 mM MgCl_2_, 5 mM D-glucose, 10 mM HEPES, 10 mM CaCl_2_, pH = 7.4 (NaOH).

### Recording procedures for patch clamp

To acquire current-voltage (I-V) relationships of sodium channels in DRG cells, test potentials ranged from -120 to +100 mV in 10 mV steps from a holding potential of -120 mV using EPC-10 (HEKA, Germany). For the activation curve of sodium channels, test potentials ranged from -80 to +100 mV in 5 mV steps from a holding potential of -120 mV. For the inactivation curve of sodium channels, test potentials ranged from -120 to +40 mV in 5 mV steps from a holding potential of -120 mV for 1000 ms, then stepped to 0 mV for 50 ms, and finally stepped to -120 mV for 150 ms. For the recovery curve of sodium channels, test potential was stepped to -10 mV for 5 ms to inactivate the sodium current, then stepped to -120 mV for different times to allow the sodium current to recover, and finally stepped to -10 mV for 50 ms to detect the sodium current. V_50_ was calculated by GraphPad Prism version 5.01 and shifts were calculated by the numerical change of V_50_ for activation and inactivation curves (Additional files 1 and 2).

To acquire current-voltage (I-V) relationships of potassium channels in DRG cells, test potentials ranged from -80 to +80 mV in 10 mV steps from a holding potential of -80 mV using EPC-10 (HEKA, Germany). 

To acquire current-voltage (I-V) relationships of calcium channels in DRG cells, test potentials ranged from -60 to +60 mV in 10 mV steps from a holding potential of -60 mV using EPC-10 (HEKA, Germany). The calcium currents of Ca_v_1.2 and Ca_v_2.2 were induced by a 400 ms depolarization of 10 mV from a holding potential of -60 mV.

Micropipettes were pulled from borosilicate glass capillary tubing (1.0-2.0 mm diameter) by using a P97 puller (Sutter Instrument Co.). After micropipettes contacting with the cell, negative pressure suction was applied to form GΩ sealing. After the formation of GΩ sealing, the fast capacitance was compensated, and then the negative pressure was applied to break the cell membrane to form the whole cell recording mode. The resistances of micropipettes were 2-5 MΩ. Slow capacitance was compensated and the membrane capacitance and series resistance (<15 MΩ) are recorded. The experimental data were collected by EPC-10 amplifier (HEKA, Germany) and analyzed in software patchmaster (HEKA, Germany).

### Data analysis

Software GraphPad Prism version 5.01 was used to statistically analyze the data and plot the curves. Data are presented as the mean values and standard deviation. For the experimental repetition times, electrophysiological experiments in DRG cells were repeated 3 times (n = 3) and electrophysiological experiments in HEK293 cells were repeated 8 times (n = 8).

## Results

### Synthesis, purification and identification of conotoxin Vr3a

**Figure 1:**
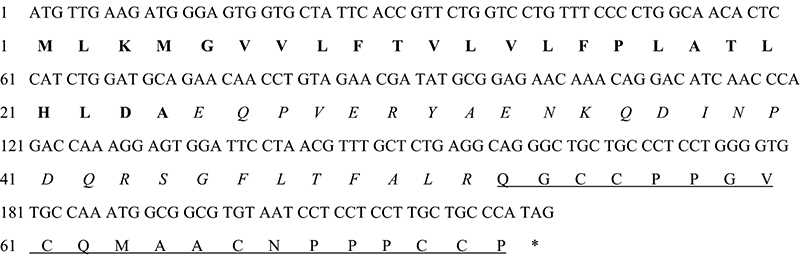
The full-length cDNA and putative amino acid sequence of Vr3a. The signal region is in bold, pro-region is in italics and mature region is underlined.

**Figure 2:**
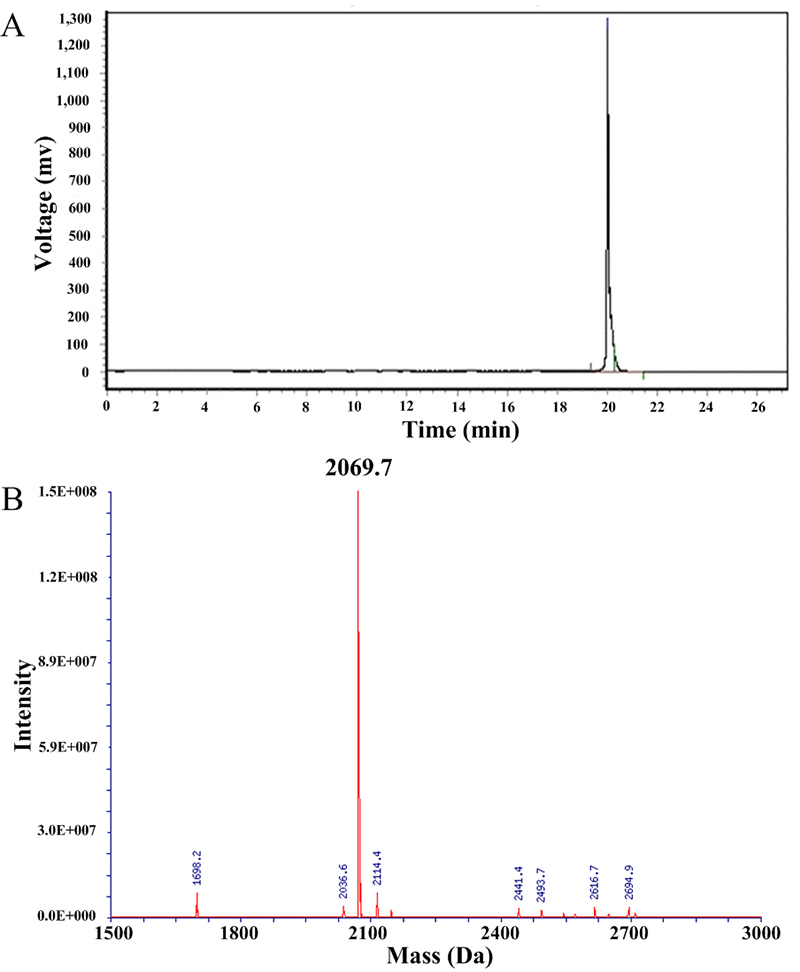
HPLC chromatogram and mass spectrum of the synthesized peptide Vr3a. **(A)** Reverse phase HPLC purification of the synthesized peptide Vr3a. The retention time is 20.03 minutes and the B% is ~60%. **(B)** Mass spectrum of the synthesized peptide Vr3a.

Vr3a gene was identified from a worm-hunting *Conus varius* by PCR. The cDNA and amino acids of Vr3a were shown in Figure 1. The predicted mature region of Vr3a contains six cysteines, which could form three pairs of disulfide bonds. According to the disulfide bonds of other M-4 and M-5 branch conotoxins [10], the mature Vr3a peptide was synthesized with three pairs of disulfide bonds (1-4, 2-5, 3-6) using a solid-phase polypeptide synthesis method. The synthesized crude peptide was purified by HPLC and identified by mass spectrometry (Figure 2A and 2B). The average mass of the synthesized peptide was 2068.7 Da, which was consistent with the molecular weight of Vr3a, indicating that Vr3a with three disulfide bonds was successfully synthesized. The monoisotopic mass spectrum of the Vr3a was shown in Additional file 3.

### Effects of Vr3a on DRG sodium, potassium and calcium currents

**Figure 3:**
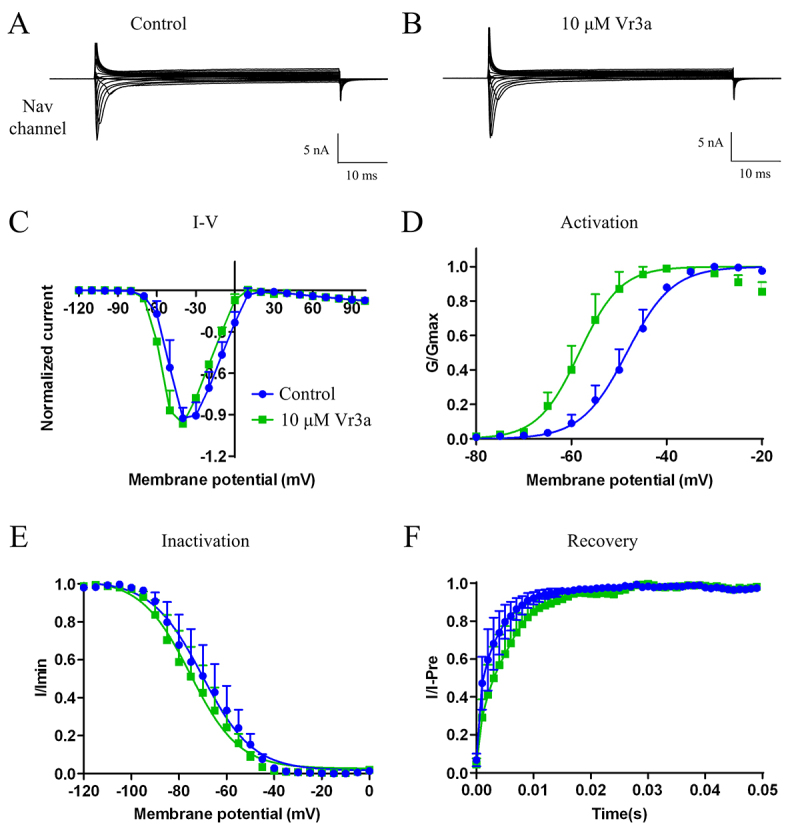
Effects of 10 μM Vr3a on sodium channel currents in rat DRG neurons. Effects of vehicle **(A)** control and **(B)** 10 μM Vr3a on sodium channel currents in rat DRG neurons. Effects of 10 μM Vr3a on **(C)** the current-voltage (I-V) relationships, **(D)** activation, **(E)** inactivation and **(F)** recovery of sodium channel currents in DRG neurons.

Vr3a was tested its electrophysiological activities on the acute isolated rat DRG cells using patch clamp. For the sodium channels, 10 μM Vr3a has no effects on the peak of the sodium currents (n = 3, Figure 3A and 3B), but can induce a ~10 mV shift in a polarizing direction in the current-voltage relationship (Figure 3C). 10 μM Vr3a can also induce a negative ~10 mV shift in the activation curve (Figure 3D) and a negative ~5 mV shift in the inactivation curve (Figure 3E). For the recovery curve, 10 μM Vr3a can induce a ~0.002 second shift in delaying sodium channel recovery (Figure 3F).

**Figure 4:**
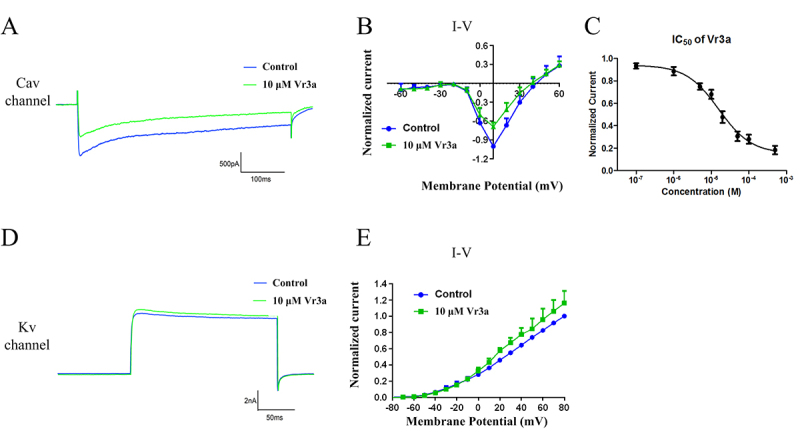
Effects of 10 μM Vr3a on potassium and calcium channel currents in rat DRG neurons. **(A)** Effects of 10 μM Vr3a on calcium channel currents in rat DRG neurons. **(B)** Effects of 10 μM Vr3a on the current-voltage (I-V) relationships of calcium channel currents in DRG neurons. **(C)** The IC_50_ value of Vr3a on calcium channel currents in rat DRG neurons. **(D)** Effects of 10 μM Vr3a on potassium channel currents in rat DRG neurons. **(E)** Effects of 10 μM Vr3a on the current-voltage (I-V) relationships of potassium channel currents in DRG neurons.

For the calcium channels in DRG cells, 10 μM Vr3a can inhibit 31.26% ± 4.53% of the peak calcium currents and do not induce a shift in the current-voltage relationship (n = 3, Figure 4A and 4B). The IC_50_ value of Vr3a on calcium channel currents in rat DRG neurons is 19.28 ± 4.32 μM (n = 3, Figure 4C). For the potassium channels in DRG cells, 10 μM Vr3a can increase 19.61 ± 5.12% of the peak potassium currents and do not induce a shift in the current-voltage relationship (n = 3, Figure 4D and 4E).

### Effects of Vr3a on human Ca_v_1.2 and Ca_v_2.2 currents

**Figure 5:**
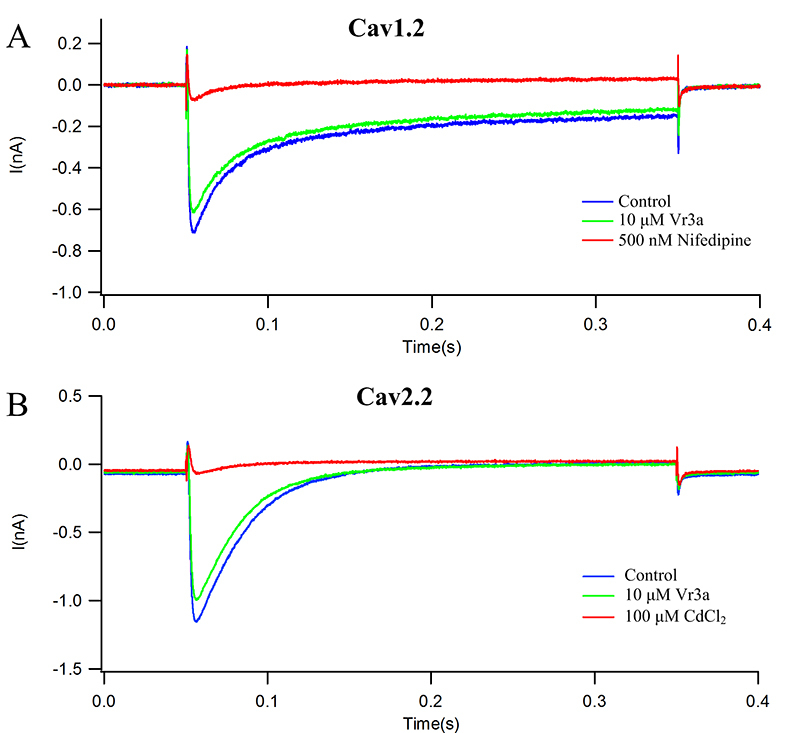
Effects of 10 μM Vr3a on human Ca_v_1.2 and Ca_v_2.2 currents in HEK293 cells. **(A)** Effects of vehicle control, positive control (500 nM nifedipine) and 10 μM Vr3a on human Ca_v_1.2 currents. **(B)** Effects of vehicle control, positive control (100 μM CdCl2) and 10 μM Vr3a on human Ca_v_2.2 currents.

Plasmids of human Ca_v_1.2 and Ca_v_2.2 were transfected into HEK293 cells respectively, and Vr3a were tested on the human Ca_v_1.2 and Ca_v_2.2. As shown in Figure 5, 10 μM Vr3a can inhibit 15.32% ± 5.41% of the human Ca_v_1.2 currents (n = 8, Figure 5A), and 500 nM Nifedipine was used as a positive control. 10 μM Vr3a can inhibit 12.86% ± 4.93% of the human Ca_v_2.2 currents (n = 8, Figure 5B), and 100 μM CdCl2 was used as a positive control.

## Discussion

Conotoxins that effect on sodium, potassium or calcium channels can be used as neuropharmacology research tools or drug leads. For the sodium channels, µ- and µO-conotoxins, which specifically target different voltage-gated sodium channel subtypes, can be used as analgesic compounds for pain therapy [19]. Among the nine functional Na_v_ subtypes, Na_v_1.7 and Na_v_1.8 were mainly involved in inflammatory pain, neuropathic pain and transduction of nociceptive information [20,21]. Therefore, the Na_v_1.7 inhibitor µ-conotoxin SxIIIC and the Na_v_1.8 inhibitors µ-conotoxin TsIIIA, µO-conotoxin MrVIB, and MfVIA are promising drugs for treating pain [22-25]. For the potassium channels, the conotoxin inhibitors are used in neuroprotection, cardioprotection or anticancer [26-28]. κM-conotoxin RIIIK, which inhibits human K_v_1.2, can reduce ischemia/reperfusion-induced infarction in rats [29,30]. For the calcium channels, the conotoxin inhibitors of calcium channels are used in the treatment of absence seizures, chronic pain or neurological disorders [31]. The most famous conotoxin, ω-conotoxin MVIIA, which inhibits Ca_v_2.2 currents, is used clinically for treating chronic pain [32]. According to the results in this study, Vr3a may be used as a drug lead of analgesics for its calcium current inhibitory activity, but its additional sodium channel shift activity and potassium current increase activity may introduce additional side effects or other unknown physiological effects, which should be studied in depth in the future.

Unlike the µ-conotoxins that inhibit sodium currents, Vr3a does not inhibit sodium currents but can induce a ~10 mV shift in the current-voltage relationship, suggesting that Vr3a may be a gating modifier that interact with the voltage-sensing domains of Na_v_ channels [33]. Similar to the ω-conotoxin that inhibit calcium currents, Vr3a inhibit calcium currents and does not induce a shift in the current-voltage relationship, suggesting that Vr3a may occupy the binding pocket to block calcium channels and does not interact with the voltage-sensing domains [34]. Unlike the κM-conotoxin that inhibit potassium currents, Vr3a can increase the potassium currents and the mechanism needs further study, which may be related to cell excitability [35].

Several conotoxins have been reported to target two different ion channels. For example, µ-conotoxin CnIIIC, an inhibitor of Na_v_1.2 and Na_v_1.4, can also inhibit neuronal nicotinic acetylcholine receptors [36]. A J-superfamily conotoxin pl14a can inhibit K_v_1.6 and nicotinic acetylcholine receptors [37]. An O-superfamily conotoxin SO3 can inhibit both neuronal sodium and potassium currents in cultured rat hippocampal neuron [38]. Two conotoxins MrVIA and MrVIB can both affect sodium and calcium currents in Lymnaea neurons [39]. In addition to conotoxins, other peptide toxins have also been reported to target multiple ion channels. For example, two spider toxins, Tap1a and Tap2a, can inhibit Na_v_ and Ca_v_3 channels at nanomolar to micromolar concentration [40]. With the expansion of ion channel research, more toxins like Vr3a targeting different types of ion channels may be discovered in the future. 

Vr3a (QGCCPPGVCQMAACNPPPCCP) is a proline-rich M-superfamily conotoxin from the worm-hunting Conus varius. Six of its 21 amino acids are proline, especially four proline at the C-terminal. Vr3a has no sequence similarity with conotoxins from other species, and may be derived from conotoxins of the same *Conus* species by amino acid insertion as the third loop of Vr3e, Vr3d, Vr3c, Vr3b and Vr3a is L, SP, DPP, NPP and NPPP (Table 1). We speculate that an evolutionary selection pressure for some kind of physiological functions makes the toxin accumulate proline. However, proline is not a key amino acid for some conotoxins affecting sodium channels, potassium channels and calcium channels, such as κM-RIIIJ affecting K_v_1.2 channels [30], µO-MfVIA affecting Na_v_1.8 channels [41] and ω-MVIIC affecting calcium channels [42]. Therefore, whether Vr3a has other physiological functions or proline has unknown interactions with sodium channels, potassium channels or calcium channels need to be studied in the future research. Different from other target-specific conotoxins with nanomolar effective concentration, the effects of Vr3a on sodium, potassium or calcium currents are weak even at 10 µM, whether Vr3a affects other calcium subtypes or other ion channels needs further research.

**Table 1:** Comparison of Vr3a and other typical M-superfamily conotoxins.

Name	GenBank number	Signal region	Pro-region	Mature region
**M-superfamily conotoxins from *Conus varius***				
Vr3e	AEX60121.1	MLKMGVVLFIFLVLFPLATLQLDA	DQPVERYAENKQLLNTDERREIILSALR	-RQ **CC** DSNS **C** EYPK **C** L--- **CC** NG
Vr3d	AEX60202.1	MLKMGVVLFTFLVLFPLATLQLDA	DQPVERNAENKQDINPDERRGFITLALR	HRG**CC**PIGP**C** LQSV **C** SP-- **CC** P-
Vr3c	AEX60204.1	MLKMGVVLFTFLVLFPLARLQLDA	DQPVERNAENKQDINPDERKRFLTLALR	QGR **CC**PYGP**C** RLSM **C** DPP **CC** A-
Vr3b	AEX60198.1	MLKMGVVLFTFLVLFPLATLQLDA	DQPVERYAENKQDINPDERKAFITLALG	QEG**CC**PSGP**C** HFAA **C** NPP- **CC** T-
Vr3a	AEX60199.1	MLKMGVVLFTVLVLFPLATLHLDA	EQPVERYAENKQDINPDQRSGFLTFALR	QG**CC**PPGV **C** QMAA **C** NPPP **CC**P-
**M-superfamily conotoxins from other *Conus* species**				
tx3a	Q9BH73	MLKMGVVLFIFLVLFPLATLQLDA	DQPVERYAENKQLLSPDERREIILHALG	TR**CC**SWDV**C**DHPS**C**T**CC**G
mr3e	Q5EHP3	MLKMQVVLFIVLVLFPLATLQLDA	DQPVERYAENKRLLNPDERRGIILHALG	GRV**CC**PFGG**C**HEL**C**Y**CC**DG
Tx3b	P0C1N8	MSKLGALLTICLLLFSLTAVPLDGDQH	ADQPAQRLQDRIPTEDHPLFDPN	KR**CC**PPVA**C**NMG**C**KP**CC**G
GIIIA	P01523	MMSKLGVLLTICLLLFPLTALPMDGDEP	ANRPVERMQDNISSEQYPLFEKR	RD**CC**TPPKK**C**KDRQ**C**KPQR**CC**AGR
SIIIA	Q86DU6	MMSKLGVLLTVCPLLFPLTALPPDGDGP	ADRPAEEMQDDISSDEHPLFDKR	QN**CC**NGG**C**SSKW**C**RDHAR**CC**GR

For the M-superfamily conotoxins from *Conus varius*, the nonconservative amino acids are marked in red and the amino acids in the third cysteine loop are marked in green. Cysteines in the mature region are in bold for all the conotoxins.

## Conclusions

In summary, this study provides an identification of a novel three-target conotoxin, Vr3a. An amount of 10 μM Vr3a can induce a ~10 mV shift in the current-voltage relationship of sodium channels, increase 19.61% ± 5.12% of the peak potassium currents, and inhibit 31.26% ± 4.53% of the peak calcium currents in rat DRG cells. In addition, 10 μM Vr3a can inhibit 15.32% ± 5.41% of the human Ca_v_1.2 currents and 12.86% ± 4.93% of the human Ca_v_2.2 currents. This novel M-superfamily conotoxin Vr3a, which has three physiological targets and no sequence homology with all other conotoxins, is a new addition to the research on application of conotoxins.

### Abbreviations

 Acm: acetamidomethyl; DRG: dorsal root ganglion; FDA: Food and Drug Administration; I-V: current-voltage; MALDI-MS: matrix assisted laser desorption ionization mass spectrum; Mmt: methoxytriphenyl; SD: Sprague-Dawleys; Trt: triphenylmethyl.

## Supplementary material

The following online material is available for this article:

Additional file 1. Effects of 10 μM Vr3a (Vr3-NPPP01) on the activation of sodium channel currents in DRG neurons.

Additional file 2. Effects of 10 μM Vr3a (Vr3-NPPP01) on the inactivation of sodium channel currents in DRG neurons.

Additional file 3. Monoisotopic molecular mass spectrum of Vr3a.
