# Supplementary material for: A novel proline-rich M-superfamily conotoxin that can simultaneously affect sodium, potassium and calcium currents
Source: J Venom Anim Toxins Incl Trop Dis. 2021 Jun 11;27:e20200164. doi: 10.1590/1678-9199-JVATITD-2020-0164 (PMC8230863; doi:10.1590/1678-9199-JVATITD-2020-0164)
Supplement: Supplementary file 1 [file 1678-9199-jvatitd-27-e20200164-s1.pdf]

J Venom Anim Toxins incl Trop Dis

https://doi.org/10.1590/1678-9199-JVATITD-2020-0164

Supplementary Material to "A novel proline-rich M-superfamily conotoxin that can simultaneously affect sodium, potassium and calcium currents"

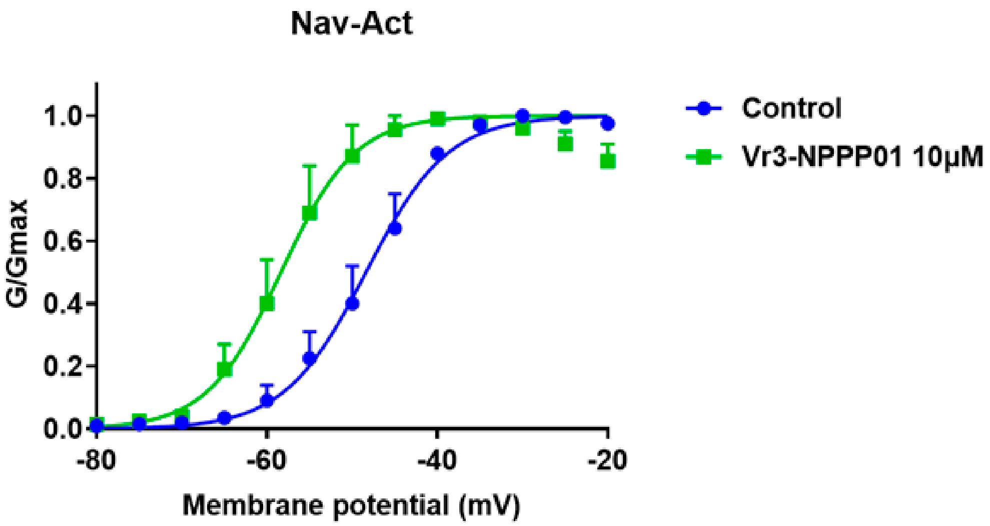

	Control	Vr3-NPPP01 10μM
Best-fit values		
Bottom	= 0.000	= 0.000
Top	= 1.000	= 1.000
V50	-48.37	-58.35
Slope	4.809	4.335
Std. Error		
V50	1.113	2.156
Slope	0.9803	1.899

Additional file 1. Effects of 10 µM Vr3a (Vr3-NPPP01) on the activation of sodium channel currents in DRG neurons.
