# Supplementary material for: A novel proline-rich M-superfamily conotoxin that can simultaneously affect sodium, potassium and calcium currents
Source: J Venom Anim Toxins Incl Trop Dis. 2021 Jun 11;27:e20200164. doi: 10.1590/1678-9199-JVATITD-2020-0164 (PMC8230863; doi:10.1590/1678-9199-JVATITD-2020-0164)
Supplement: Supplementary file 2 [file 1678-9199-jvatitd-27-e20200164-s2.pdf]

J Venom Anim Toxins incl Trop Dis

https://doi.org/10.1590/1678-9199-JVATITD-2020-0164

Supplementary Material to "A novel proline-rich M-superfamily conotoxin that can simultaneously affect sodium, potassium and calcium currents"

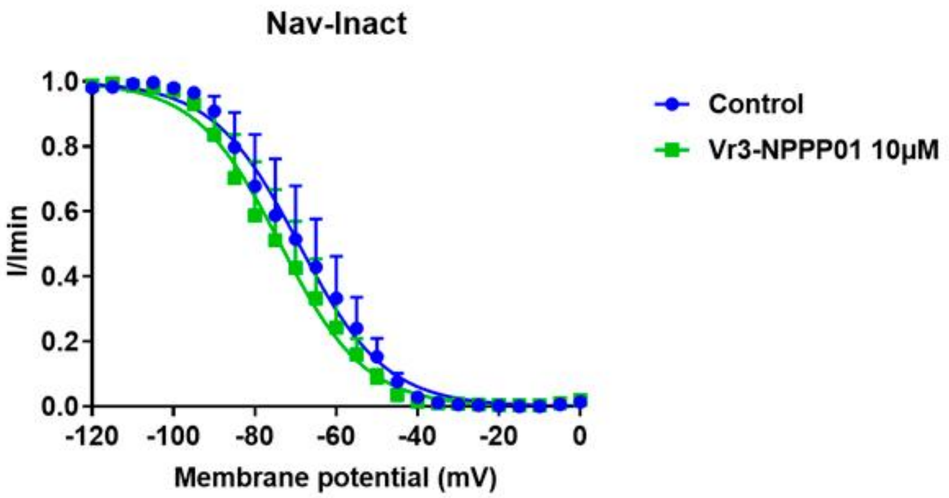

	Control	Vr3-NPPP01 10μM
Best-fit values		
Bottom	= 0.000	= 0.000
Top	= 1.000	= 1.000
V50	-69.19	-73.74
Slope	-10.68	-10.42
Std. Error		
V50	1.100	1.021
Slope	0.9698	0.9015

Additional file 2. Effects of 10 μM Vr3a (Vr3-NPPP01) on the inactivation of sodium channel currents in DRG neurons.
