# Supplementary material for: A novel proline-rich M-superfamily conotoxin that can simultaneously affect sodium, potassium and calcium currents
Source: J Venom Anim Toxins Incl Trop Dis. 2021 Jun 11;27:e20200164. doi: 10.1590/1678-9199-JVATITD-2020-0164 (PMC8230863; doi:10.1590/1678-9199-JVATITD-2020-0164)
Supplement: Supplementary file 3 [file 1678-9199-jvatitd-27-e20200164-s3.pdf]

*J Venom Anim Toxins incl Trop Dis*

https://doi.org/10.1590/1678-9199-JVATITD-2020-0164

# Supplementary Material to “A novel proline-rich M-superfamily conotoxin that can simultaneously affect sodium, potassium and calcium currents”

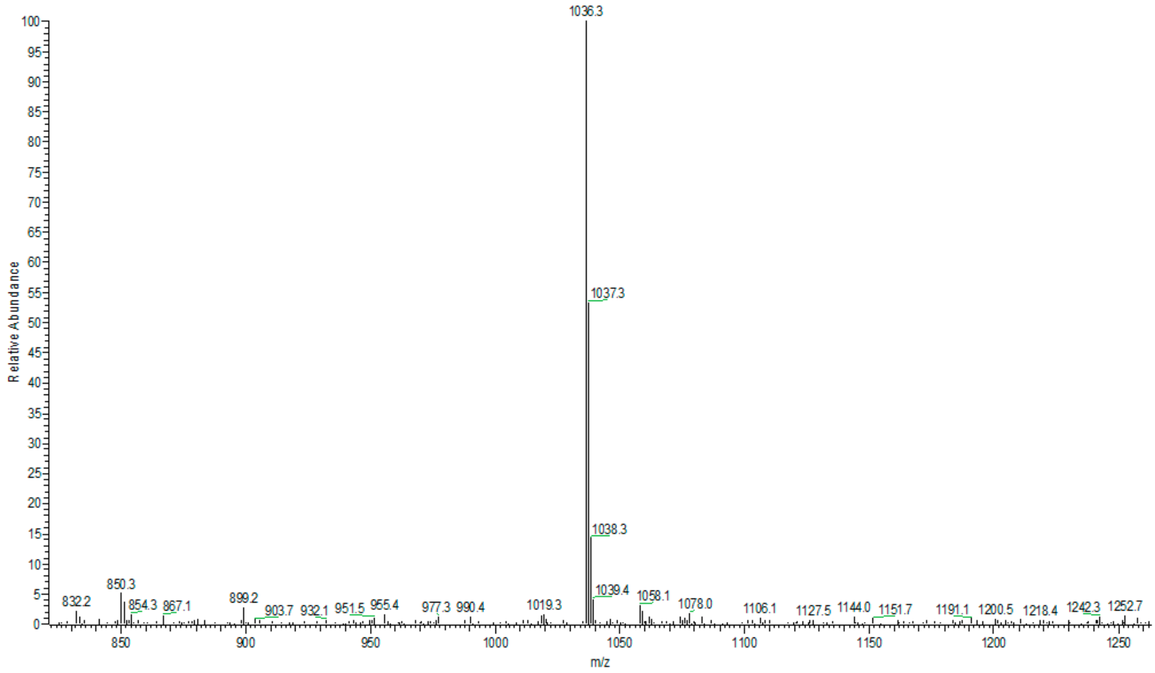


**Additional file 3.** Monoisotopic molecular mass spectrum of Vr3a.
